# Beyond Wellens’ Syndrome: Two Cases of Dynamic Electrocardiographic (ECG) Changes in Critical Left Anterior Descending Coronary Artery (LAD) Occlusion

**DOI:** 10.7759/cureus.115119

**Published:** 2026-08-24

**Authors:** Nikolay N Nikolov, Rozen K Grigorov, Stefan N Yambolov, Yanko G Yankov

**Affiliations:** 1 Department of Medicine, Medical University "Prof. Dr. Paraskev Stoyanov", Varna, BGR; 2 Department of Interventional Cardiology, University Multi-profile Hospital for Active Treatment "St. Marina", Varna, BGR; 3 Department of Cardiology and Rheumatology, Medical University "Prof. Dr. Paraskev Stoyanov", Varna, BGR; 4 Department of Maxillofacial Surgery, University Multi-profile Hospital for Active Treatment "St. Marina", Varna, BGR; 5 Department of General and Operative Surgery, Medical University "Prof. Dr. Paraskev Stoyanov", Varna, BGR

**Keywords:** acute coronary syndrome, coronary angiography, electrocardiographic pattern, left anterior descending artery, myocardial ischemia, percutaneus coronary intervention, wellens’ syndrome

## Abstract

Wellens’ syndrome represents a high-risk electrocardiographic (ECG) manifestation of critical left anterior descending coronary artery (LAD) disease, characterized by dynamic T-wave changes that may evolve over time and precede extensive anterior myocardial infarction. In this report, we describe two patients with critical LAD disease who presented with dynamic ECG changes consistent with different Wellens’ syndrome patterns, highlighting the variability of this condition. The first was a 68-year-old male patient who presented with chest pain and dyspnea. His initial ECG showed transient ST-segment elevation in the anterior leads, which later evolved into biphasic T waves, consistent with a Wellens’ type A pattern. The second patient, a 62-year-old man, presented with acute chest pain and diaphoresis. Early ECG changes included transient ST-segment elevation, followed by the development of deep T-wave inversions in the precordial leads, consistent with a Wellens’ type B pattern. In both cases, cardiac biomarkers were elevated, while ECG findings were either subtle or non-diagnostic in the early phase. Coronary angiography confirmed critical LAD involvement, and both patients underwent successful percutaneous coronary intervention.

## Introduction

Wellens’ syndrome is a well-described electrocardiographic pattern first reported in the early 1980s by de Zwaan, Wellens and colleagues in patients with unstable angina and characteristic anterior T-wave abnormalities. Their original observations linked this pattern to critical proximal left anterior descending coronary artery (LAD) stenosis and showed that, without timely intervention, it frequently preceded extensive anterior myocardial infarction [1,2].

Classically, Wellens’ syndrome is characterized by deeply inverted or biphasic T waves in the precordial leads, most often V2-V4, typically appearing after a transient resolution of chest pain. Cardiac biomarkers may be normal or only mildly elevated in the early phase, and patients may appear clinically stable despite the presence of critical coronary disease [3,4]. The electrographic (ECG) pattern is thought to reflect a dynamic process of transient LAD occlusion followed by spontaneous reperfusion, explaining its evolution in the absence of persistent ST-segment elevation [5].

Reported incidence varies across clinical cohorts, and Wellens’ syndrome remains an important but potentially underrecognized presentation of critical LAD disease. Recognition may be delayed because the characteristic ECG abnormalities can be subtle, transient, or appear after symptom resolution, when the patient may otherwise seem clinically stable [3,4].

Although not classified by European Society of Cardiology (ESC) guidelines as a formal ST-segment elevation equivalent, Wellens’ sign is recognized as an non-ST-elevation acute coronary syndrome (NSTE-ACS) ECG abnormality associated with severe proximal LAD disease [6]. This distinction is clinically important: in pain-free patients it identifies a high-risk pre-infarction state requiring early invasive evaluation, whereas in the presence of ongoing or recurrent ischemic symptoms it should be managed with immediate invasive assessment.

In this report, we present two patients with critical LAD disease and dynamic ECG changes corresponding to different Wellens’ patterns. The cases demonstrate distinct type A and type B phenotypes associated with dynamic coronary occlusion and differing angiographic findings, highlighting the variability of presentation and the potential for misleading or non-diagnostic findings in the early phase of acute coronary syndrome (ACS).

## Case presentation

Clinical case one

A 68-year-old man with arterial hypertension presented after two episodes of acute chest pain on the day of admission. He was 180 cm tall and weighed 85 kg, corresponding to a body mass index (BMI) of 26.2 kg/m². He had quit smoking in 2021, reported no significant alcohol consumption, and was not taking regular medication. At approximately 11:00 a.m., he experienced sudden retrosternal chest pain during physical activity, which resolved after several minutes of rest. At around 3:00 p.m., the chest pain recurred at rest, prompting the patient to call the emergency medical services. On-site evaluation revealed markedly elevated blood pressure. The patient received sublingual isosorbide dinitrate, intramuscular clonidine, and aspirin 250 mg before being transported to our institution.

**Figure 1:**
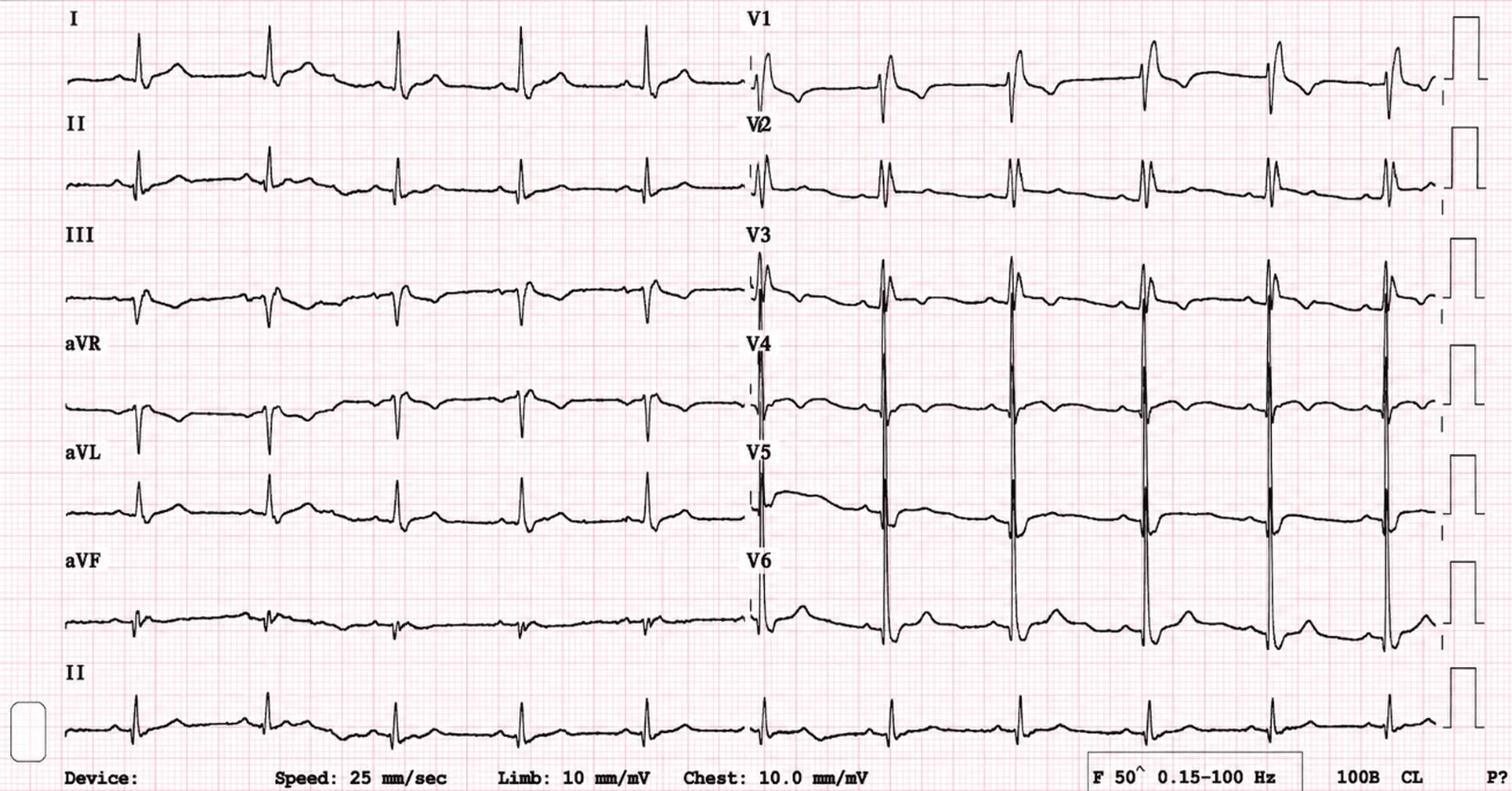
Initial electrocardiogram demonstrating sinus rhythm with complete right bundle branch block and T-wave inversions in leads V3-V5

On arrival, the patient was pain-free and hemodynamically stable, with a blood pressure of 140/80 mmHg. The initial ECG demonstrated sinus rhythm with complete right bundle branch block and T-wave inversions in leads V3-V5 (Figure 1).

Serial high-sensitivity cardiac troponin I measurements were 327.0, 329.0, 82.0, and 36.0 ng/L (reference range: 2.5-45 ng/L). Complete blood count was unremarkable, with hemoglobin 143 g/L (reference range: 125-172 g/L), a white blood cell count of 9.2 × 10⁹/L (reference range: 3.60-10.50 × 10⁹/L), and a platelet count of 282 × 10⁹/L (reference range: 140-440 × 10⁹/L). Serum creatinine was 109 µmol/L (reference range: 62-115 µmol/L), corresponding to an estimated glomerular filtration rate of approximately 66 mL/min/1.73 m². Electrolytes were within normal limits, with sodium 139 mmol/L (reference range: 132-146 mmol/L) and potassium 4.0 mmol/L (reference range: 3.50-5.50 mmol/L). The lipid profile demonstrated dyslipidemia, with total cholesterol 6.3 mmol/L (reference range: 2.70-5.20 mmol/L), low-density lipoprotein (LDL) cholesterol 4.31 mmol/L (reference range: 0-2.6 mmol/L), high-density lipoprotein (HDL) cholesterol 1.02 mmol/L (reference range: 1.0-1.6 mmol/L), and triglycerides 2.21 mmol/L (reference range: 0-1.7 mmol/L).

**Figure 2:**
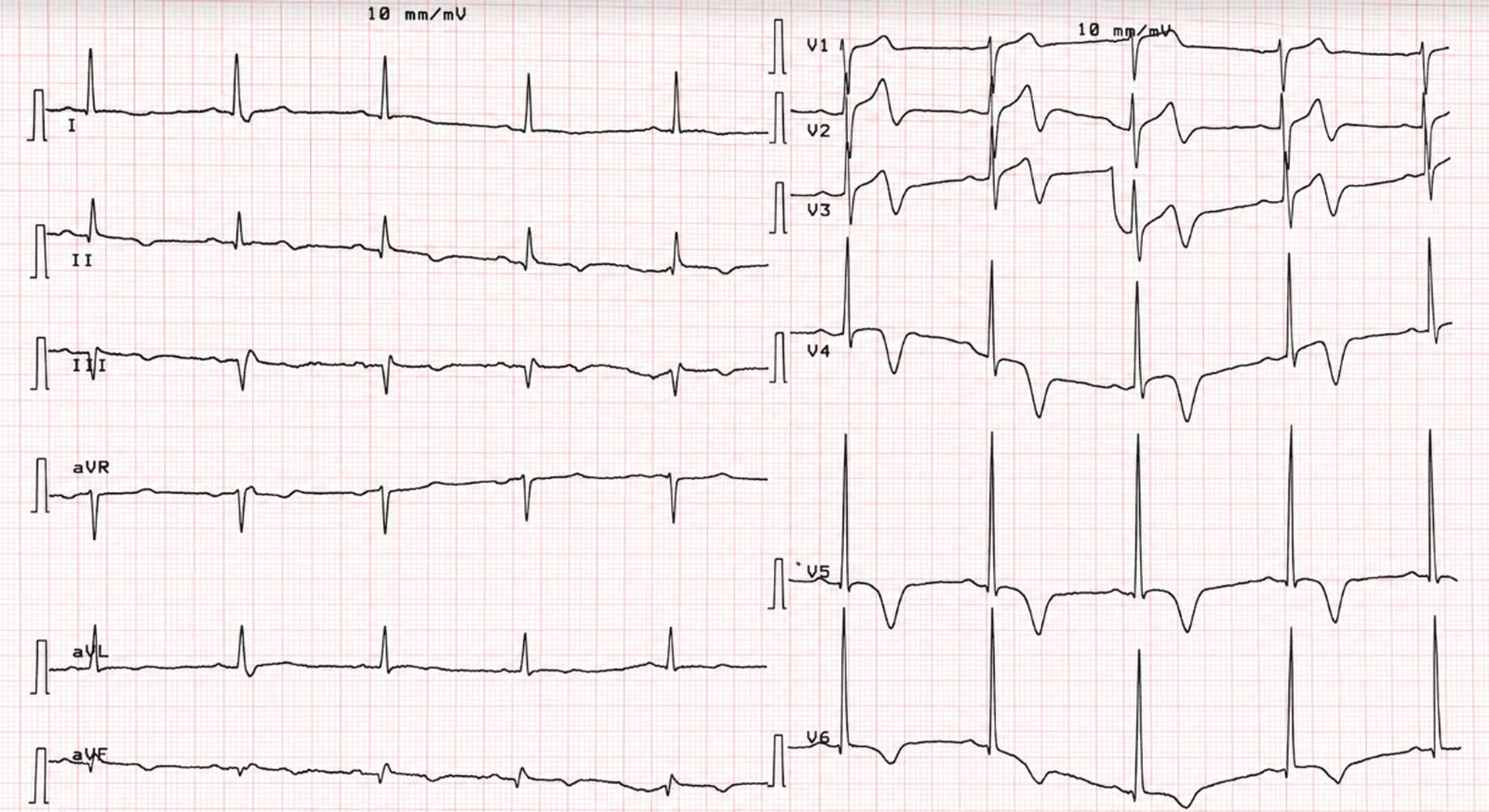
Follow-up ECG demonstrating Wellens’ type A changes with anterior ST-segment elevation, evolving anterior T-wave inversion, and associated inferior ST-segment elevation with pathological Q waves

During the second day of hospitalization, the patient developed recurrent chest pain accompanied by dynamic ECG changes. The repeat ECG demonstrated ST-segment elevation in leads V1-V3 associated with biphasic T waves, consistent with a Wellens’ type A pattern, together with deep T-wave inversions in leads V4-V6. Mild ST-segment elevation was also present in the inferior leads (Figure 2).

Transthoracic echocardiography demonstrated mildly reduced left ventricular systolic function with an estimated ejection fraction of approximately 45% and regional wall motion abnormalities involving the apical and inferior left ventricular segments. Right ventricular systolic function was preserved, no significant valvular heart disease was identified, and left ventricular filling pressures were within normal limits.

**Figure 3:**
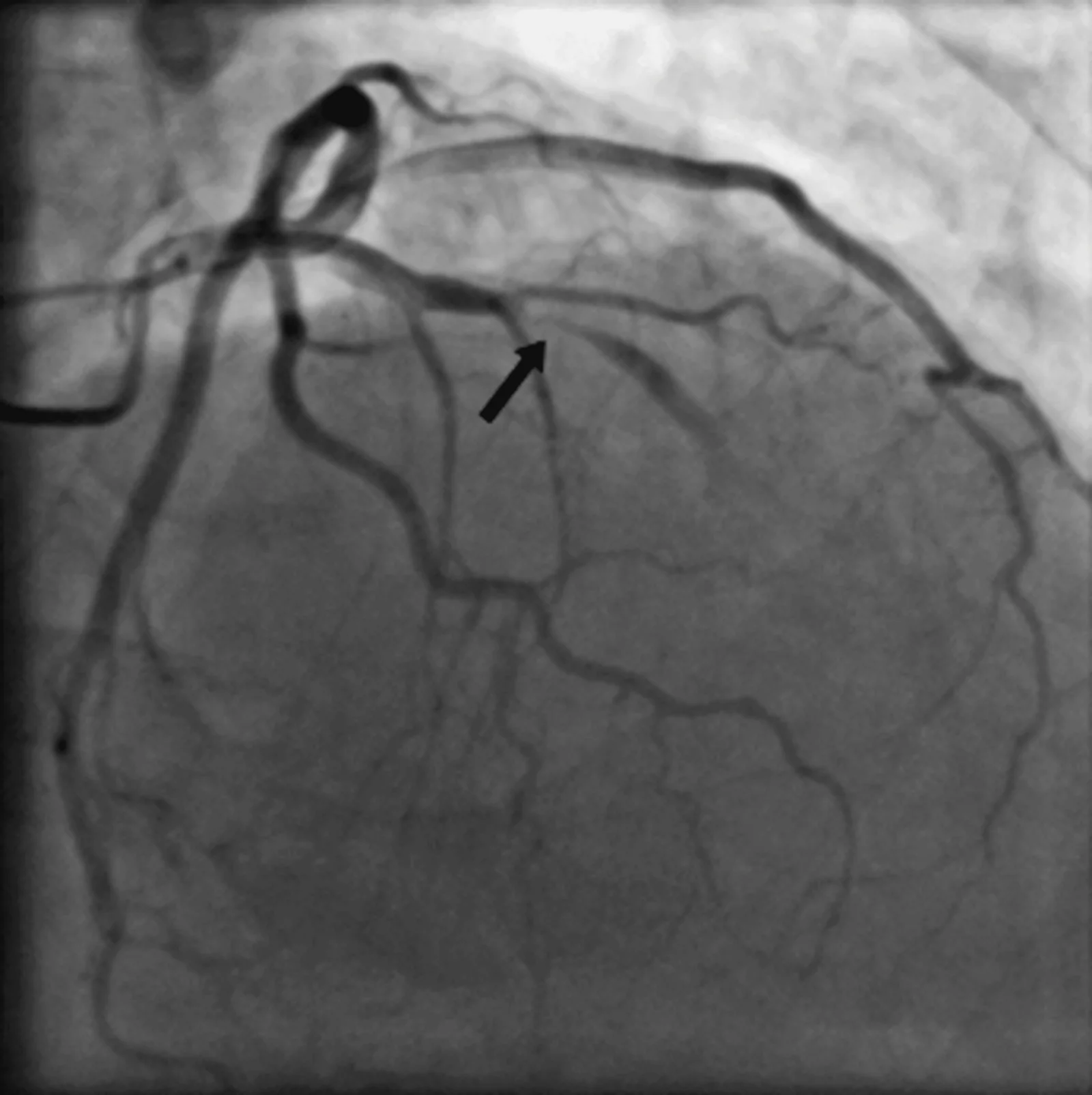
Coronary angiography demonstrating thrombotic total occlusion of the mid segment of a large wrap-around left anterior descending artery

Coronary angiography demonstrated a co-dominant coronary circulation, with the posterior descending artery arising from the right coronary artery and the posterolateral branch arising from the left coronary system. The LAD was a large type 3 wrap-around vessel, extending beyond the apex and supplying the inferoapical myocardium, with thrombotic total occlusion of the mid segment (Figure 3).

**Figure 4:**
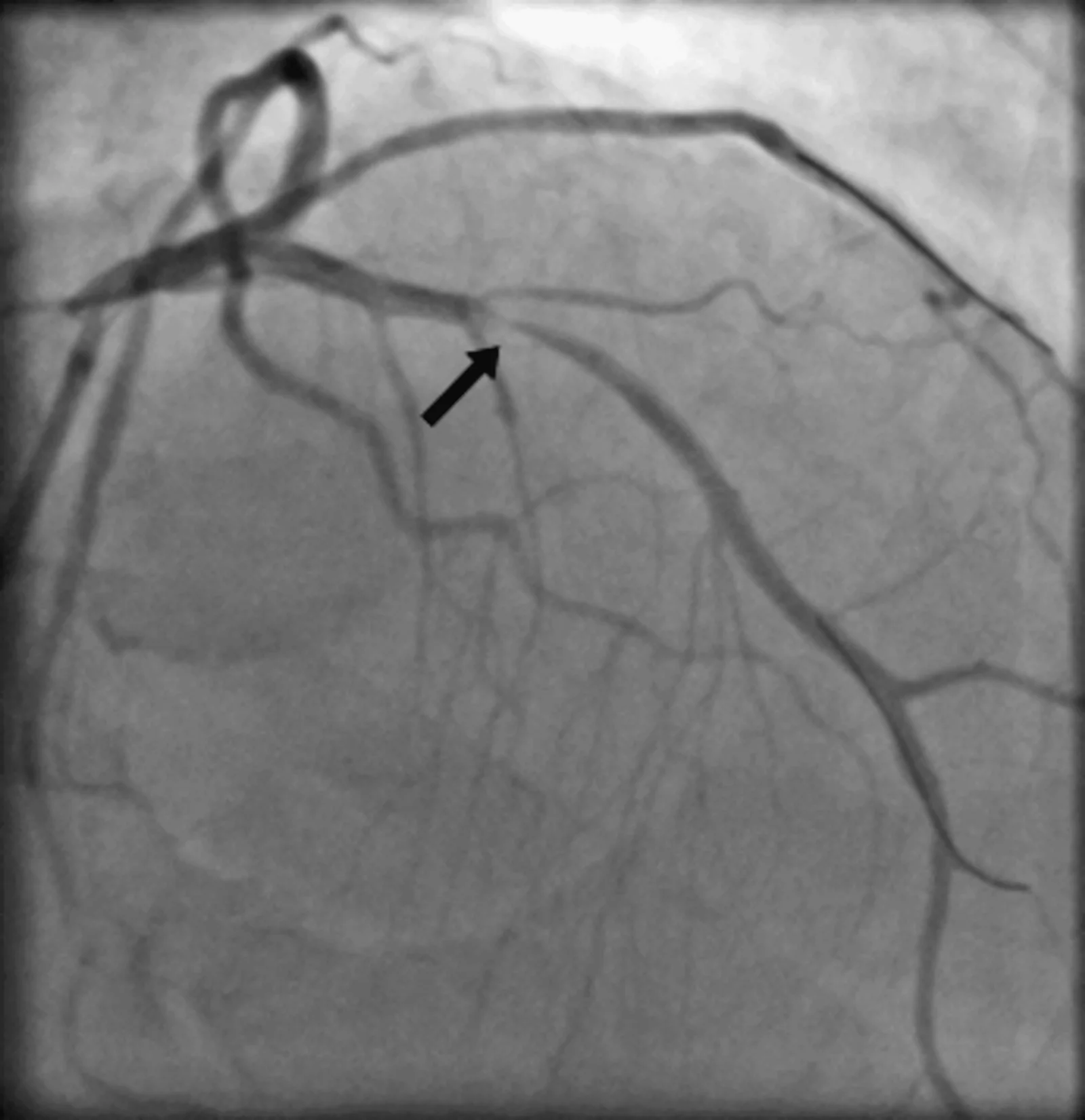
Coronary angiography demonstrating partial spontaneous recanalization of the mid left anterior descending artery following guidewire passage

The left circumflex and right coronary arteries had no significant stenoses (Figure 4).

**Figure 5:**
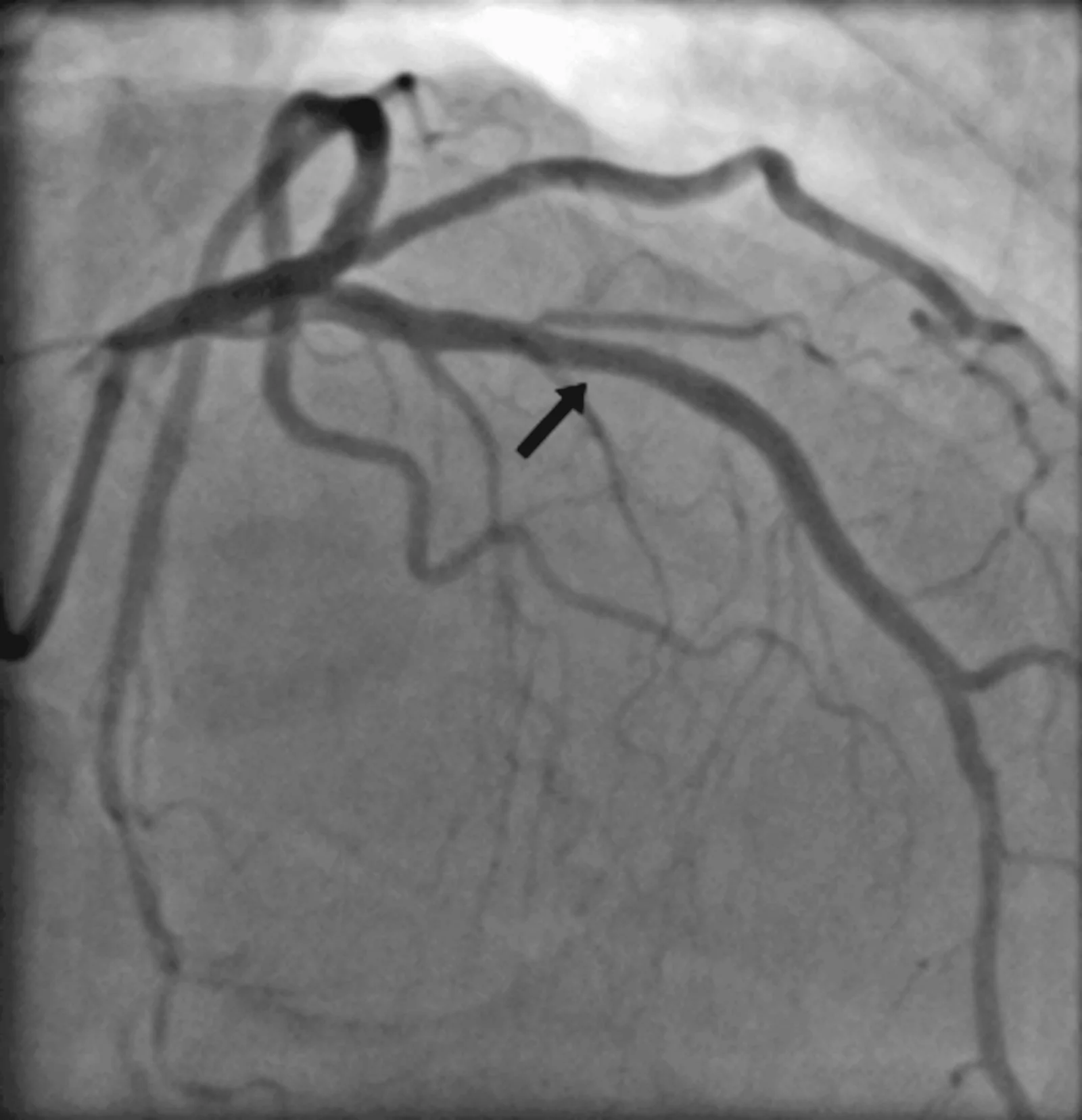
Final angiographic result after PCI of the left anterior descending artery demonstrating complete lesion coverage, restoration of TIMI grade 3 flow, and no residual stenosis

Primary percutaneous coronary intervention was performed. Introduction of the guidewire resulted in partial recanalization of the occluded mid-LAD (Figure 4). Balloon predilatation was then performed using a 2.0×20 mm balloon catheter, followed by implantation of a 3.0×26 mm sirolimus-eluting stent and proximal optimization with a 3.0×12 mm non-compliant balloon. Final angiography demonstrated an excellent angiographic result with restoration of Thrombolysis in Myocardial Infarction (TIMI) grade 3 coronary flow (Figure 5).

The subsequent in-hospital course was uneventful, with no recurrent chest pain, hemodynamic instability, bleeding, or vascular access complications. The patient was discharged in stable condition on guideline-directed secondary prevention therapy including dual antiplatelet therapy with acetylsalicylic acid and ticagrelor.

Clinical case two

A 62-year-old man with a history of arterial hypertension and dyslipidemia presented with sudden-onset severe chest pain with irradiation to both upper limbs, with diaphoresis, beginning approximately 30 minutes prior to admission.

**Figure 6:**
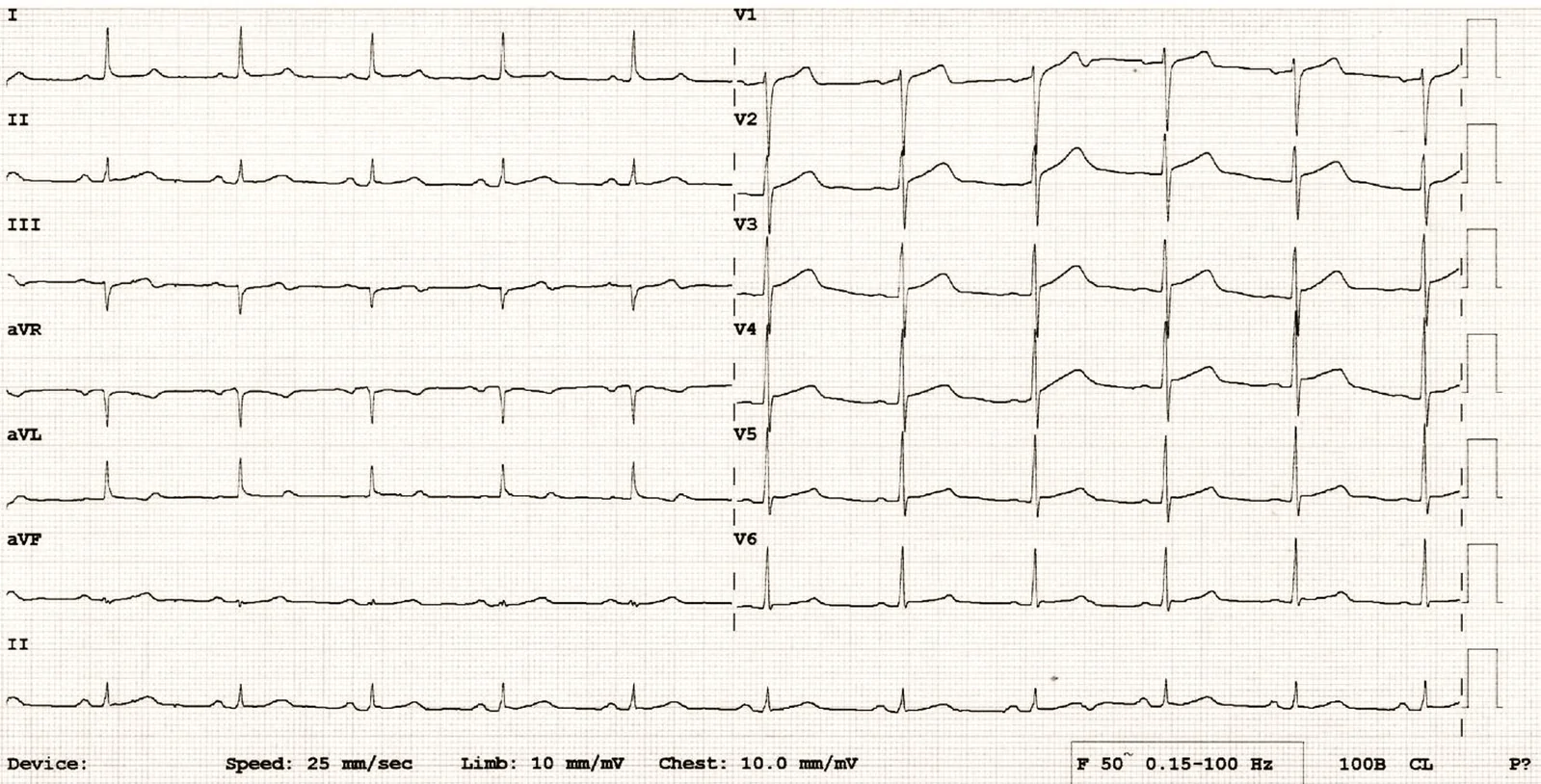
Initial electrocardiogram demonstrating sinus rhythm with subtle ST-segment elevation in the anterior leads (V1-V6)

On admission, the patient was hypertensive, with a blood pressure of 210/130 mmHg and a regular heart rhythm of approximately 80 beats per minute. The initial ECG showed subtle ST-segment elevation in leads V3-V4, without reciprocal ST-segment depression, together with broad-based T waves suggestive of ongoing anterior ischaemia. These changes did not meet conventional ST-elevated myocardial infarction (STEMI) criteria (Figure 6).

The patient was treated for ACS with intravenous glyceryl trinitrate, unfractionated heparin 5000 IU, aspirin 300 mg, and intravenous clonidine.

**Figure 7:**
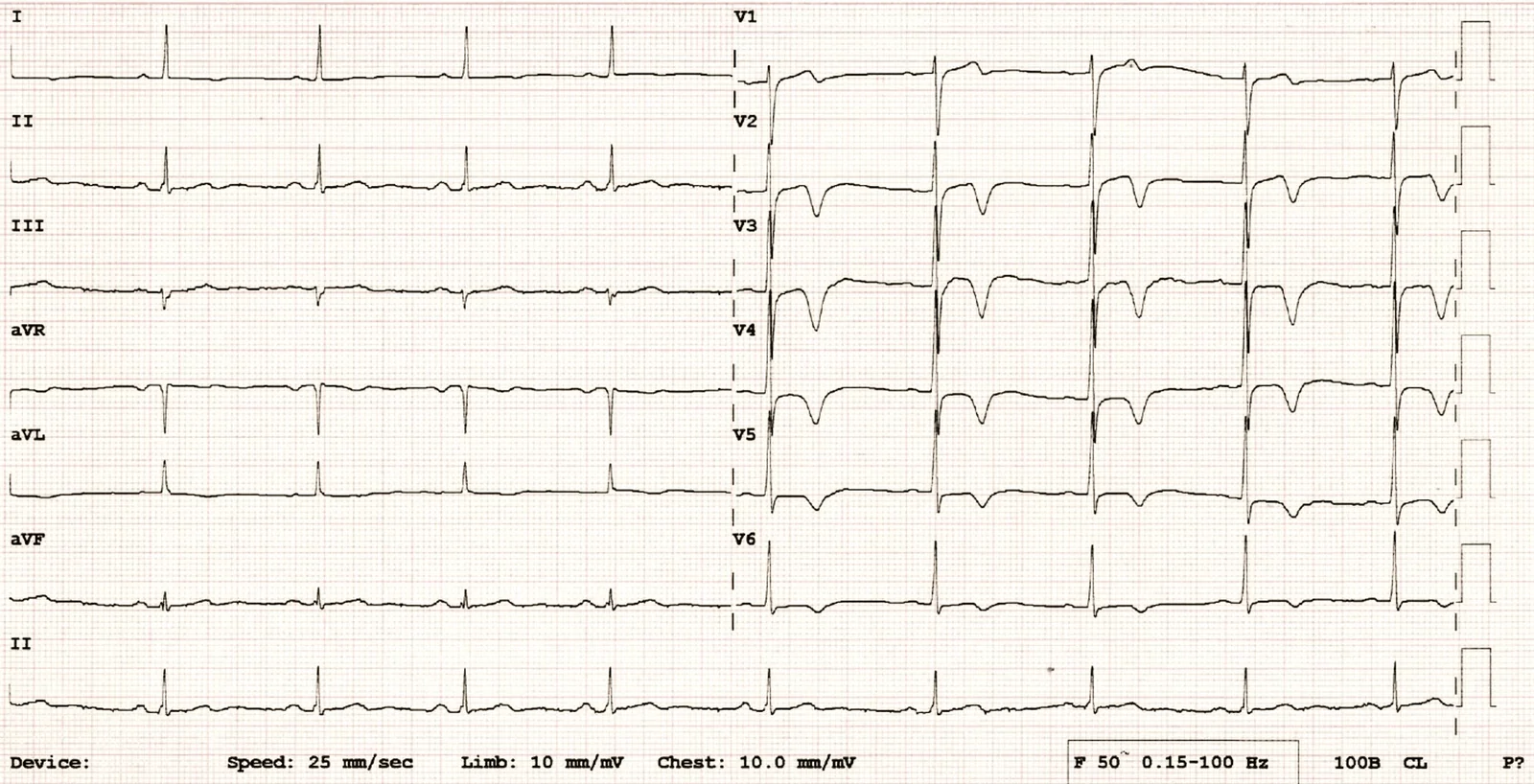
Follow-up electrocardiogram demonstrating sinus rhythm with deep, symmetric T-wave inversions in the anterior leads (V1-V6), consistent with a Wellens’ type B pattern

During the first hours of hospitalization, his symptoms partially resolved, and repeat ECGs were non-diagnostic. However, on the second day, he developed recurrent chest discomfort, and an ECG recorded during the episode demonstrated new changes, including deep, symmetric T-wave inversions in leads V1-V6, consistent with a Wellens’ type B pattern (Figure 7).

Laboratory evaluation showed a dynamic rise in cardiac biomarkers, with high-sensitivity troponin I peaking at 2717.45 ng/L (reference range 2.5-45 ng/L).

Transthoracic echocardiography showed preserved left ventricular systolic function and mild concentric left ventricular hypertrophy. Subtle regional wall motion abnormalities were noted, with discrete hypokinesia of the apical segments. No significant valvular abnormalities were identified.

**Figure 8:**
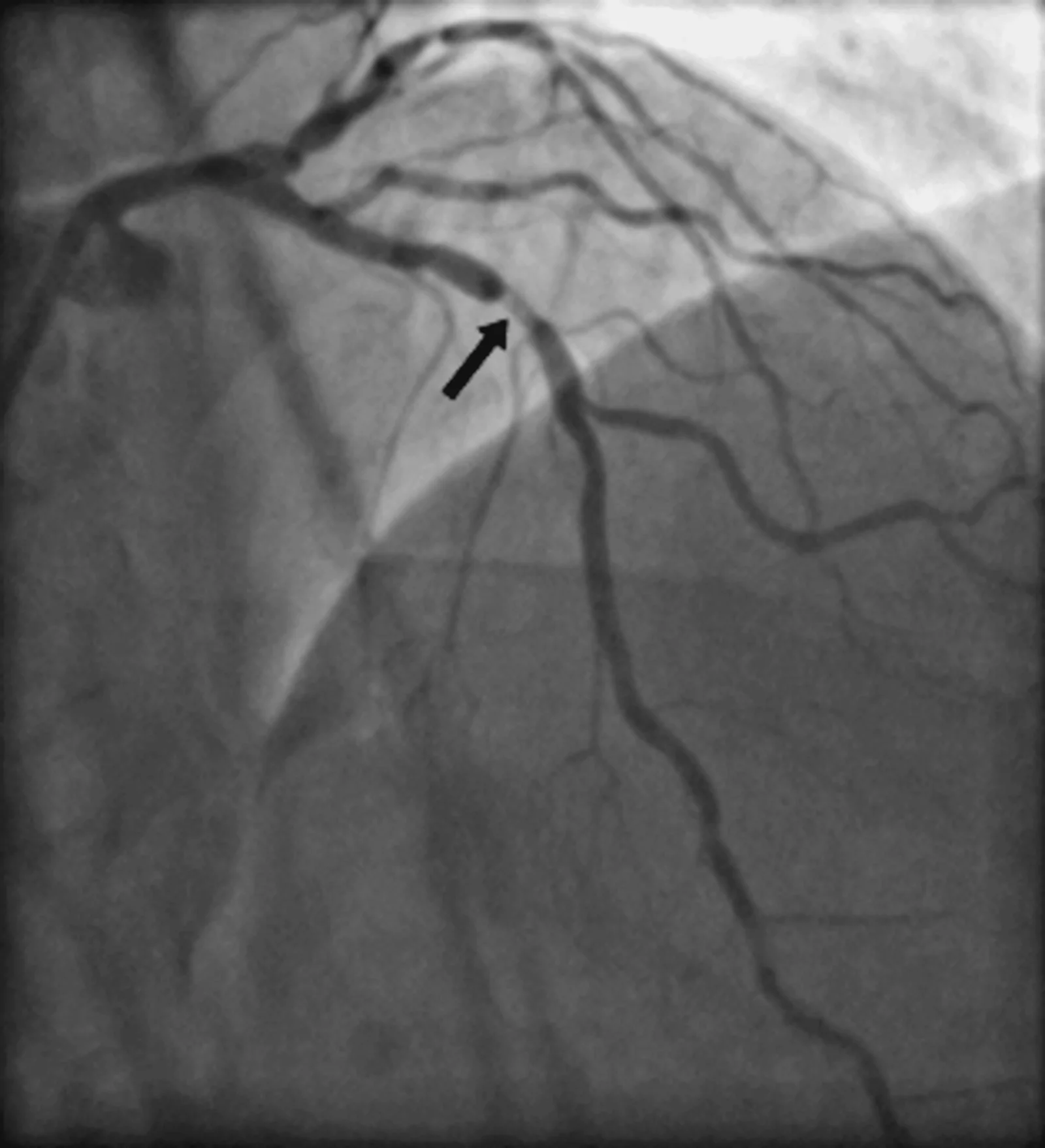
Coronary angiography demonstrating diffuse disease of the left anterior descending artery with proximal subtotal occlusion and distal significant stenosis, along with moderate distal left main narrowing and a significant mid-segment stenosis of the left circumflex

An immediate coronary angiography was performed via right radial access, which demonstrated a right-dominant coronary circulation with severe multivessel coronary artery disease. The left main showed a distal plaque with a 30% stenosis. The LAD was diffusely diseased, with a proximal subtotal occlusion with significant thrombotic burden. The left circumflex had a 70% stenosis in the mid segment (Figure 8).

**Figure 9:**
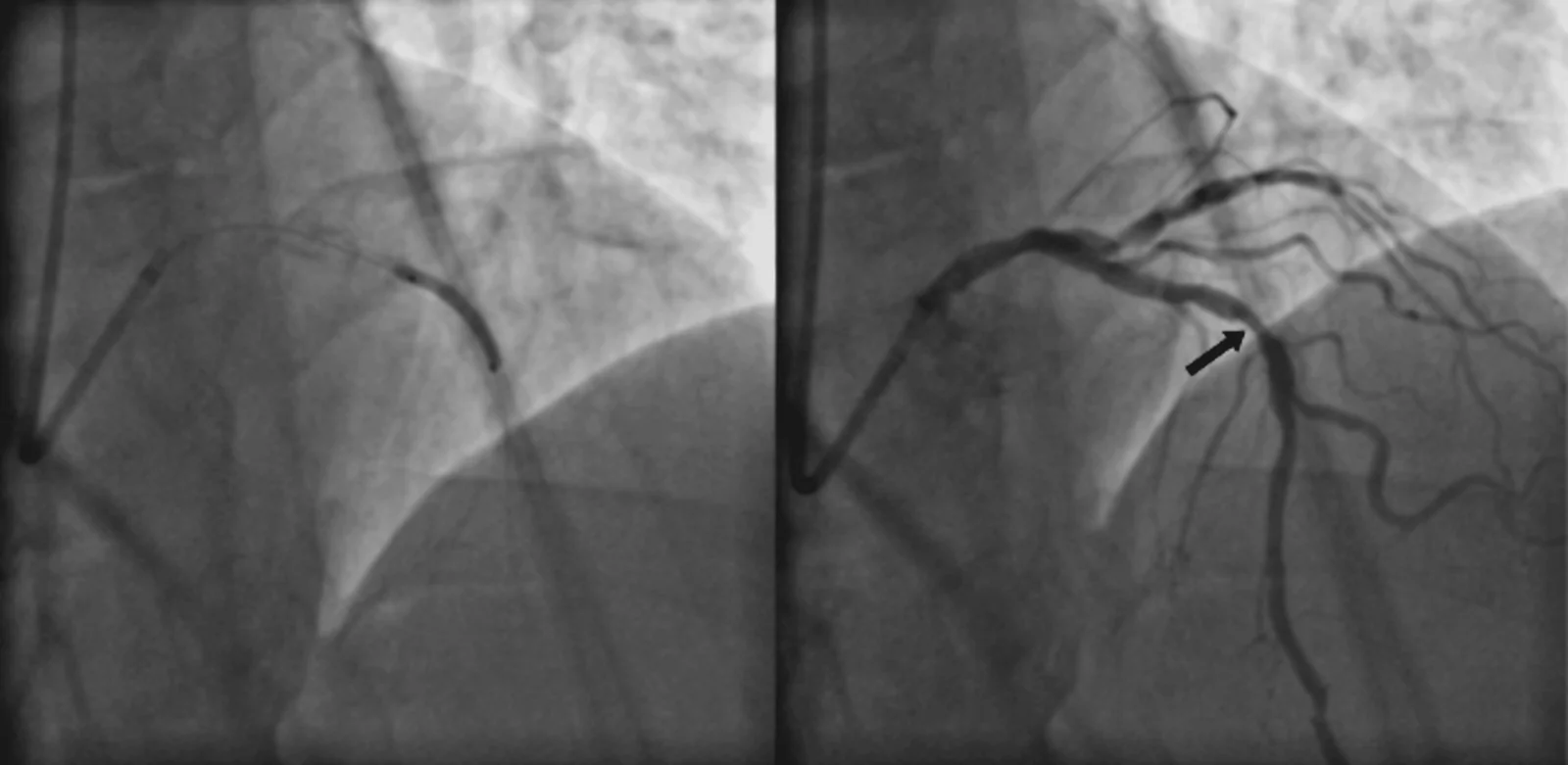
Balloon angioplasty of the left anterior descending artery Left panel: balloon positioned at the site of occlusion; Right panel: restoration of vessel patency following dilation.

The right coronary artery was also diffusely affected, with a long atherosclerotic plaque in the proximal to mid segments, resulting in up to 75% luminal narrowing (Figure 9).

**Figure 10:**
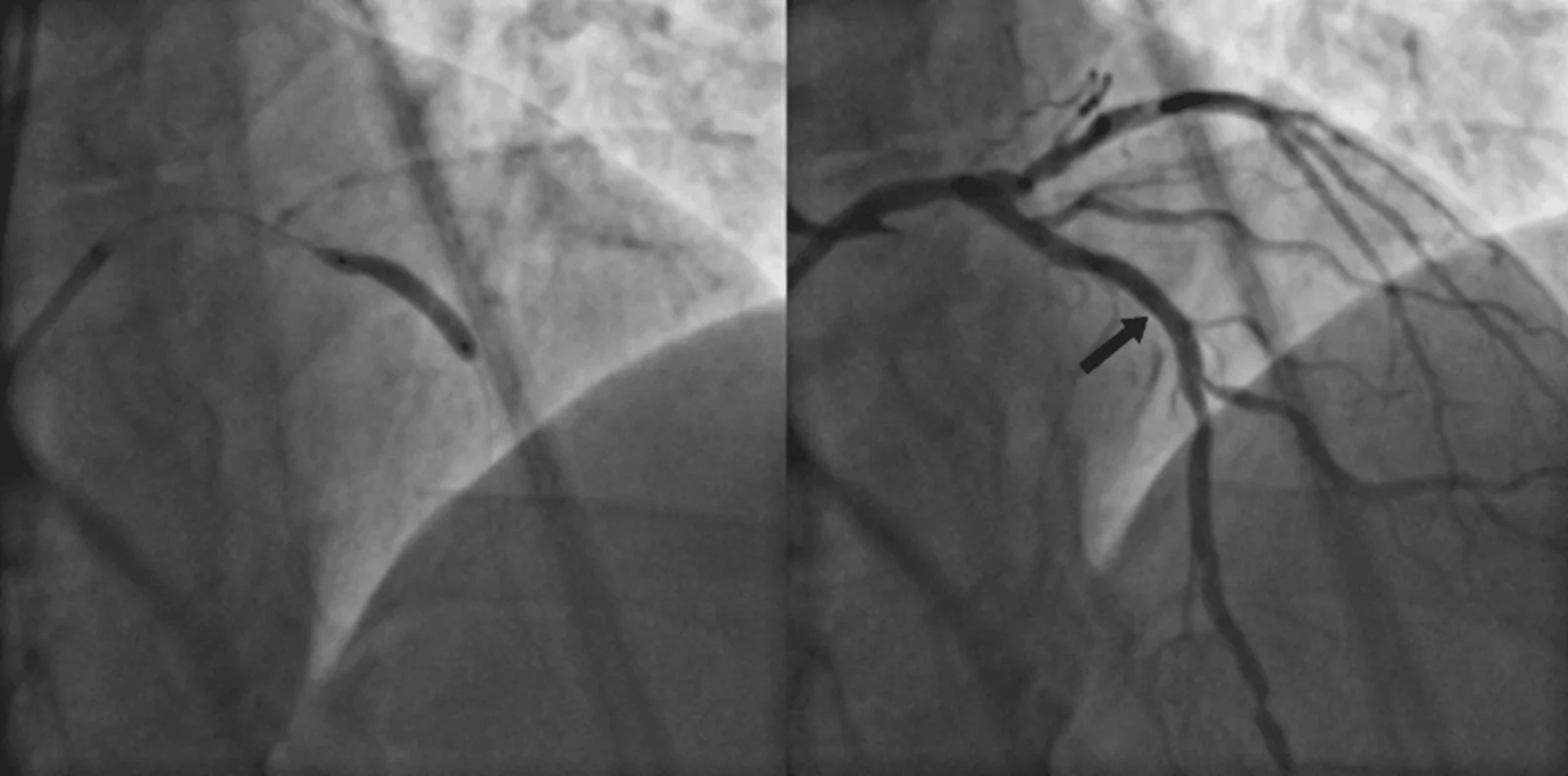
Coronary angiography showing deployment of a drug-eluting stent in the proximal LAD LAD, Left anterior descending coronary artery; Left panel demonstrates stent positioning, while the right panel shows the post-implantation result.

Percutaneous coronary intervention was performed with initial predilatation of the LAD using a 2.0×20 mm balloon (Figure 9). Although coronary angiography demonstrated severe multivessel coronary artery disease, the LAD was considered the culprit lesion based on the ECG changes involving the anterior territory, the presence of a thrombotic proximal subtotal LAD occlusion, and the associated apical wall motion abnormality on ECG. A drug-eluting stent (3.0×22 mm) was subsequently implanted in the proximal LAD, followed by post-dilatation with a 3.0×15 mm non-compliant balloon (Figure 10).

The significant left circumflex and right coronary artery lesions were considered non-culprit lesions and were therefore not treated during the index procedure. Following a discussion with the Heart Team, staged reassessment and subsequent revascularization of the remaining lesions were planned after recovery from the index event.

On follow-up ECG, three days after PCI, there was improvement in regional wall motion abnormalities. The patient had a stable in-hospital course following revascularization, with no further episodes of chest pain or rhythm disturbances. He was discharged on guideline-directed secondary prevention therapy, including dual antiplatelet therapy with acetylsalicylic acid and ticagrelor. The ECG at the three-month follow-up showed that the patient had a complete recovery of left ventricular segmental kinetics.

## Discussion

The present cases illustrate the heterogeneous clinical and electrocardiographic spectrum of LAD-related ACS presenting with Wellens’-type repolarization abnormalities. In the first patient, recurrent chest pain was accompanied by dynamic ECG evolution from anterior T-wave inversion to a Wellens’ type A pattern with transient ST-segment elevation, while the second patient developed a classic Wellens’ type B pattern following initial transient ST-segment elevation and subsequent symptom recurrence. Despite the differences in ECG morphology, both patients had dynamic ischemic symptoms, elevated cardiac biomarkers, and critical LAD occlusion requiring urgent PCI. These cases emphasize that Wellens-type patterns represent high-risk ischemia and should not be interpreted as benign ECG findings.

In a cross-sectional study of patients with ACS, Wellens’ criteria were identified in 15% of cases, with both type A and type B patterns represented and with angiographic variation involving proximal LAD, mid-LAD, multivessel disease, and even non-obstructive coronary findings [7,8].

A central feature of these cases is the presence of two different Wellens’ ECG phenotypes leading to the same high-risk anatomical diagnosis. Type A Wellens’ pattern is classically characterized by biphasic T waves, most often in the anterior precordial leads, whereas type B is characterized by deep, symmetric T-wave inversion, which may extend across the precordial leads [3,7]. Type B changes are reported more frequently, while type A changes may be more subtle and therefore easier to overlook [3,7]. Importantly, these patterns should not be regarded as separate clinical entities, but rather as part of a dynamic ECG spectrum that may reflect changing coronary flow conditions in the LAD territory [9].

This concept is well illustrated by the two patients in the present report. Although the ECG morphology differed, both patients had critical LAD occlusion confirmed by coronary angiography. In the first patient, the angiographic findings also explained the coexistence of anterior and inferior ECG abnormalities, as the wrap-around anatomy of the LAD supplied both the apical and inferior myocardial segments. Recent reports have similarly shown that Wellens-type changes may reflect subtotal or even complete LAD occlusion, supporting their interpretation as a dynamic occlusion-reperfusion phenotype rather than a marker of stable critical stenosis alone [7,10].

Recognition of both type A and type B patterns is clinically important because either may indicate unstable proximal LAD disease. Type A changes may be subtle after symptom resolution, whereas type B changes may be mistaken for non-specific T-wave inversion. Failure to recognize either pattern can delay invasive treatment, a critical issue given that 75% proportion of medically treated patients in the original Wellens’ series progressed to extensive anterior myocardial infarction [1,11].

From a pathophysiological perspective, Wellens-type repolarization abnormalities are considered to reflect transient LAD occlusion followed by spontaneous reperfusion, rather than persistent transmural ischemia [1,3]. This mechanism explains both the absence of conventional STEMI criteria and the high-risk nature of the ECG pattern. The 2023 ESC guidelines describe Wellens’ sign among NSTE-ACS ECG abnormalities associated with severe proximal LAD disease, rather than as a formal ST-segment elevation equivalent [6]. Nevertheless, in the presence of ongoing or recurrent ischemic symptoms, such patients should be considered very high risk and managed with immediate invasive assessment, PCI if needed, and optimal secondary prevention therapy [12].

## Conclusions

This report highlights the clinical importance of recognizing Wellens-type ECG changes as a dynamic and evolving manifestation of critical LAD disease rather than a static ECG pattern. The appearance of a Wellens’ T-wave pattern after resolution of chest pain should not be interpreted as a reassuring sign. Instead, it may represent spontaneous reperfusion following a critical LAD occlusion, reflecting an unstable occlusion-reperfusion process that remains associated with a high risk of recurrent ischemia and extensive anterior myocardial infarction. In patients with recurrent or ongoing ischemic symptoms, Wellens’-type changes should trigger immediate invasive evaluation, even in the absence of persistent ST-segment elevation, as timely recognition and revascularization can directly influence outcomes and prevent progression to extensive myocardial injury. For clinicians, recognition of these dynamic ECG patterns in routine practice is essential, as even subtle or transient changes may identify patients with critical LAD disease.
